# Artificial Intelligence in Nursing Practice Education: A Systematic Review and Meta‐Analysis

**DOI:** 10.1155/jonm/6626865

**Published:** 2026-09-10

**Authors:** Jingting Wang, Mingxi Kuang, Jiayuan Chen, Youqing Yan, Zhen Li

**Affiliations:** ^1^ Department of Organ Transplantation, The First Affiliated Hospital of Kunming Medical University, Kunming 650032, China, kmmc.cn

**Keywords:** application effect, artificial intelligence, meta-analysis, nursing practice, systematic review

## Abstract

**Objective:**

To systematically evaluate the application effect of artificial intelligence (AI) technology in nursing practice courses and to provide evidence‐based guidance for the intelligent reform of nursing education.

**Methods:**

Computerized searches were performed in PubMed, Web of Science, Embase, the Cochrane Library, CNKI, WanFang, CBM, and VIP databases for randomized controlled trials and quasi‐experimental studies on the educational effectiveness of AI in nursing practice courses. The retrieval period was from the establishment of each database to February 2026. Data extraction, quality evaluation, and literature screening were carried out separately by two researchers. RevMan 5.4 was used to conduct the meta‐analysis. The PROSPERO registration number for this review protocol is CRD420261403298. The PRISMA 2020 guidelines are followed in this systematic review.

**Results:**

A total of 16 studies with a sample size of 1647 participants were included, including 7 randomized controlled trials and 9 quasi‐experimental studies. The results of this meta‐analysis indicate that incorporating AI into nursing practical training programs may enhance students′ learning outcomes in a variety of ways, with beneficial facilitative effects noted in knowledge level (SMD = 0.72, 95% CI [0.20–1.24], *p* = 0.007), practical ability (SMD = 1.25, 95% CI [0.50–2.00], *p* = 0.001), critical thinking and clinical reasoning abilities (SMD = 0.43, 95% CI [0.21–0.65], *p* < 0.001), learning satisfaction (SMD = 0.38, 95% CI [0.16–0.59], *p* = 0.0005), and ethical awareness and decision‐making capacity (SMD = 0.50, 95% CI [0.29–0.71], *p* < 0.001). However, high heterogeneity was observed for knowledge level (*I* ^2^ = 91%) and practical ability (*I* ^2^ = 95%), while the remaining outcomes showed low to mild heterogeneity (*I* ^2^ < 50%). The overall methodological quality of the included studies was moderate to high.

**Conclusion:**

There are encouraging advantages to using AI in practical nursing courses. According to preliminary data, it may have positive benefits on a variety of learning parameters. These results are still preliminary and need to be confirmed by future high‐quality research due to significant variation, especially in knowledge level and practical abilities, as well as limitations in the quantity and design of included studies.

**Implications for Nursing Management:**

Nursing education management can gain multifaceted practical insights from the use of AI in practical training courses: (1) Institutions shall actively adopt mechanisms for the implementation, evaluation, and continuous improvement of AI‐assisted teaching; allocate resources in a coordinated manner; and advance the intelligent upgrading of practical courses to ease the shortage of clinical practice resources. (2) To improve nursing educators’ competence in AI instructional design and ethical standards, a tiered and categorized training system will be implemented. To promote educational innovation, corresponding reward and assessment schemes should be developed. (3) Teaching management must incorporate a multifaceted evaluation system for AI teaching outcomes. Formative assessment using platform data can be used to continuously monitor and enhance the quality of instruction. (4) The goals of nursing talent training should include AI digital literacy. To avoid an over‐reliance on technology, instructors must cultivate students’ critical thinking and autonomous judgment while enhancing their practical skills. (5) To standardize educational data management, a strong ethical and safety framework for AI application must be refined. This ensures a uniform and long‐lasting intelligent change of nursing education by striking a balance between humanistic care and technology empowerment.

## 1. Introduction

Nursing education is under unparalleled revolutionary pressure due to changing global population demographics and growing healthcare demands. In the meantime, practical new solutions are provided by the quick development of artificial intelligence technology. The necessity of incorporating digital competences, such as AI literacy, into health education curricula is emphasized by global health policies. In order to educate medical professionals for the digital transition, educational systems must change and adapt, according to international organizations like the World Health Organization and the International Council of Nurses [1]. Future nurses must possess strong digital literacy and human–machine collaboration skills, according to the global nursing education community [2]. In this regard, China’s Healthy China 2030 agenda, which closely follows international reform trends, places a high priority on technology‐driven healthcare services. Nursing education urgently needs to transition from knowledge transfer to competency development due to the ongoing implementation of the Healthy China 2030 goal and the digital transformation of healthcare delivery models [3]. Nursing practice courses develop students′ clinical reasoning, technical proficiency, and humanistic care skills as a vital link between theory and clinical practice. However, due to constraints including a lack of standardized patient resources and few possibilities for clinical placement, traditional teaching finds it difficult to meet the expectations of individualized and exact instruction [4]. In recent years, the breakthrough development of generative AI has provided new technical support for the transformation of nursing education [5]. Because of their natural language interaction, intelligent content creation, and real‐time feedback capabilities, large language models like ChatGPT and DeepSeek have shown distinct advantages in the field of education [6]. Previous research has examined the usefulness of AI in clinical skills, simulated consultations, and individualized learning support in practical courses such as adult nursing, pediatric nursing, basic nursing, and nursing ethics [7–10].

One of the fundamental foundations enabling the use of AI in practical nursing instruction is constructivist learning theory. This learner‐centered philosophy places more emphasis on students’ active investigation, learning, and creation of knowledge than on their passive absorption of information [11]. The adoption of AI tools further facilitates the creation of personalized and adaptive experiential learning environments to meet nursing students’ learning demands [11, 12]. According to McCoy et al., medical education must foster doctors’ ability to work with AI, which includes acknowledging the limitations of model outputs, retaining appropriate skepticism, and incorporating AI‐generated data into particular clinical settings [12]. Consequently, constructivism’s emphasis on situational creation, cooperative communication, and active knowledge construction offers essential instructional design underpinnings for AI‐powered practical nursing instruction. The majority of research designs are single‐center, small‐sample quasi‐experimental studies that lack systematic evidence synthesis based on large‐sample, multicenter randomized controlled trials (RCTs), despite the fact that previous studies have tentatively confirmed the application potential of AI technology in nursing practice teaching [13]. Additionally, studies vary greatly in terms of AI tool types, training length, and result measures, and there are currently no recognized uniform intervention paradigms or evaluation standards [14]. Therefore, in order to provide an evidence‐based basis for the intelligent reform of nursing education, a systematic review is required to thoroughly summarize the application effects of AI technology in nursing practice courses, synthesize existing evidence, and clarify its impacts on nursing students’ knowledge level, practical ability, critical thinking, and learning satisfaction. The PROSPERO registration number for this review protocol is CRD420261403298. The PRISMA 2020 guidelines are followed in this systematic review.

## 2. Materials and Methods

### 2.1. Search Strategy

PubMed, Web of Science, Embase, The Cochrane Library, CNKI (China National Knowledge Infrastructure), Wanfang Data, CBM (Chinese Biomedical Literature Database), and VIP Database are among the databases that were searched. Subject terms and free words were combined for the search, which ran from the databases’ creation until February 2026. Additionally, manual retrieval was used. We used the snowballing strategy to search the literature and searched trial registries, such as ClinicalTrials.gov. The search terms include “Students Nursing/Nursing student/Pupil nurses/Nurse student/Nurse/Nursing trainees,” “Artificial Intelligence/AI/ChatGPT/Machine learning/Deep learning/Large language model/Natural language processing,” and “Experimental teaching/Nursing experiment/Training/Simulation teaching/Simulated operation/Skill training/High fidelity simulation/Scenario simulation/OSCE.” Detailed search terms are shown in Table 1.

**TABLE 1:** English search strategy—taking PubMed as an example.

No.	Search terms	Number of literature
#1	Students, Nursing [Mesh]	34,923
#2	Nursing student [Title/Abstract] OR Pupil nurses [Title/Abstract] OR Nurse student [Title/Abstract] OR Nurse [Title/Abstract] OR Nursing trainees [Title/Abstract]	164,631
#3	#1 OR #2	190,671
#4	Artificial Intelligence [Mesh]	266,789
#5	AI [Title/Abstract] OR ChatGPT [Title/Abstract] OR Machine learning [Title/Abstract] OR Deep learning [Title/Abstract] OR Large language model [Title/Abstract] OR Natural language processing [Title/Abstract]	350,807
#6	#4 OR #5	483,198
#7	Experimental teaching [Title/Abstract] OR Nursing experiment [Title/Abstract] OR Training [Title/Abstract] OR Simulation teaching [Title/Abstract] OR Simulated operation [Title/Abstract] OR Skill training [Title/Abstract] OR High fidelity simulation [Title/Abstract] OR Scenario simulation [Title/Abstract] OR OSCE [Title/Abstract]	708,186
#8	#3 AND #6 AND #7	197

### 2.2. Inclusion and Exclusion Criteria for Literature

Literature inclusion criteria were formulated according to the PICOS principle for systematic reviews: *Population*: Nursing students (including undergraduate nursing students and pre‐licensure nursing residents); *Intervention*: Nursing practice teaching assisted by AI technology; *Comparison*: Traditional teaching mode or non‐AI‐assisted instruction; *Outcome*: Knowledge level, practical ability, critical thinking, clinical reasoning ability, etc. *Study Design*: RCTs or quasi‐experimental studies. Data were complete, and the two groups were comparable at baseline. Exclusion criteria for included literature: Publications with unavailable full texts or ineffective data extraction; duplicate publications; studies with a pre‐post self‐controlled design, conference abstracts, reviews, etc.; non‐Chinese and non‐English publications.

### 2.3. Literature Screening and Data Extraction

EndNote software was used to screen the literature. Data extraction and literature screening were carried out independently by two researchers who had received comprehensive training in evidence‐based nursing. First, duplicate publications were eliminated. A full‐text review was used for a second screening after the first screening, which involved reading the titles and abstracts of the studies. A third researcher was consulted to settle any disputes, and the final included studies were identified. Literature screening, including full‐text and title/abstract screening, was carried out separately by two researchers; 0.89 was the computed Cohen’s kappa coefficient. Three papers in all, or 1.5% of the full‐text screened literature, needed to be evaluated by a third researcher. General information about the included studies (author, publication year, study location, study design), participant characteristics (type of participants, sample size), teaching content and methods (intervention group, control group), intervention duration, and outcome measures were all included in the data extraction process.

### 2.4. Literature Quality Assessment

The quality of the included literature was independently evaluated by two researchers who had passed the exam and finished standardized evidence‐based nursing training. Cross‐checking was done on the evaluation results. A third‐party expert settled any disagreements between the two researchers. Five dimensions of bias are included in the Cochrane Handbook’s risk of bias assessment tool for RCTs [15]. The Joanna Briggs Institute (JBI) Evidence‐Based Health Care Center’s nine‐item quality rating criteria were used for quasi‐experimental research [16]. Classification of Quasi‐Experimental Studies: Grade A: All 9 criteria are fully satisfied. Grade B: The number of items marked “No” or “Unclear” is no more than 3. Grade C: All items are marked “No” or “Unclear.”

### 2.5. Statistical Methods

RevMan 5.4 was used for analysis and to create forest plots. The *I* ^2^ statistic and *p* value were used to evaluate statistical heterogeneity between separate studies. If no substantial statistical heterogeneity was found (*p* > 0.05 and *I* ^2^ < 50%), a fixed‐effects model was used to determine the pooled effect size. On the other hand, if significant heterogeneity was found (*p* < 0.05 and *I* ^2^ > 50%), a random‐effects model was employed. Only continuous variables were included in this analysis, and different outcome indicator measuring techniques were used in the available literature. Consequently, the 95% confidence interval (CI) and standard mean difference (SMD) were employed for the display. Subgroup analysis, sensitivity analysis, and descriptive analysis were carried out if there was notable clinical heterogeneity between the trials. Publication bias was evaluated using funnel plots. Statistical significance was defined as a *p* value of less than 0.05.

### 2.6. Classification of Artificial Intelligence Interventions

This study preclassified the included AI interventions into the following two categories based on the mechanisms and teaching functions of AI tools. Table 2 displays comprehensive classification findings.

1. Conversational large language models, such as ChatGPT, DeepSeek, Doubao, Gemini, and ions, support clinical reasoning and offer real‐time feedback.
2. Virtual patient simulation systems (such as the SimMom simulator and interactive VR patients): Create virtual clinical scenarios, concentrate on teaching clinical decision‐making and operational skills, and facilitate experiential learning through repeated practice in a controlled and safe setting.

**TABLE 2:** Basic characteristics of included literature (*n* = 16).

**First author (year)**	**Study region**	**Study type**	**Study population**	**Sample size (experimental/control)**	**Teaching content**

Guo et al. (2025) [7]	China	RCT	Nursing undergrad	109 (55/54)	Nursing psychology
Shin et al. (2024) [8]	South Korea	QE	Nursing undergrad	99 (52/47)	Pediatric nursing
Saatçi et al. (2024) [9]	Turkey	RCT	Nursing undergrad	180 (91/89)	Fundamentals of nursing
Arkan et al. (2025) [17]	Turkey	RCT	Nursing undergrad	96 (48/48)	Nursing process education
Abuadas et al. (2025) [18]	Jordan	QE	Nursing undergrad	115 (57/58)	Nursing ethics
Li et al. (2024) [19]	China	QE	Nursing resident	62 (32/30)	Fundamentals of nursing
Zhao (2023) [20]	China	RCT	Nursing undergrad	185 (96/89)	Nursing ethics
Molu (2024) [21]	Turkey	QE	Nursing undergrad	70 (35/35)	Pediatric nursing
Song et al. (2025) [10]	China	QE	Nursing undergrad	132 (66/66)	Thinking and communication
Park (2025) [22]	South Korea	QE	Nursing undergrad	72 (38/34)	Labor simulation for obstetric nursing
Simsek‐Cetinkaya (2023) [23]	Turkey	RCT	Nursing undergrad	103 (52/51)	Obstetric and gynecologic nursing
Fung et al. (2025) [24]	China, Thailand, Spain	RCT	Nursing undergrad	44 (22/22)	Critical care nursing
Kestel et al. (2025) [25]	Turkey	RCT	Nursing undergrad	79 (38/41)	History taking
Han et al. (2022) [26]	South Korea	QE	Nursing undergrad	61 (30/31)	Obstetric and gynecologic nursing
Liaw et al. (2024) [27]	Singapore	QE	Nursing undergrad	147 (60/87)	Immersive clinical simulation
Başaran (2024) [28]	Turkey	QE	Nursing undergrad	93 (31/31/31)	Obstetric and gynecologic nursing

**First author (year)**	**Teaching method**		**Intervention duration**	**Outcome measures**	**AI tool category**
	**Experimental**	**Control**			

Guo et al. (2025) [7]	GAI‐enabled interdisciplinary collaborative learning model	Traditional teaching model	8 credit hours	①⑦	Conversational large language models
Shin et al. (2024) [8]	AI‐assisted instruction (ChatGPT)	Traditional teaching model	30 min	②③⑧	Conversational large language models
Saatçi et al. (2024) [9]	AI‐assisted instruction (ChatGPT, Gemini, Copilot)	Traditional teaching model	5days	⑨	Conversational large language models
Arkan et al. (2025) [17]	AI‐integrated nursing process training (ChatGPT)	Standard nursing process education	4 weeks	②④⑤	Conversational large language models
Abuadas et al. (2025) [18]	AI‐assisted ethics education	Traditional teaching model	3 days	⑤⑧	Conversational large language models
Li et al. (2024) [19]	Students interact with AI for interactive learning.	Traditional teaching model	90 min	①③	Conversational large language models
Zhao (2023) [20]	AI‐assisted instruction (DeepSeek)	Traditional teaching model	16 weeks	①④	Conversational large language models
Molu (2024) [21]	AI‐assisted instruction (ChatGPT)	Traditional teaching model	4 weeks	①	Conversational large language models
Song et al. (2025) [10]	Traditional teaching + AI‐assisted instruction (Doubao)	Online‐offline hybrid teaching	24 credit hours	③⑧	Conversational large language models
Park (2025) [22]	High‐fidelity simulation + AI tutor‐assisted teaching (Danbee.ai platform)	Traditional high‐fidelity simulation training (SimMom Simulator)	5 weeks	①②③⑩	Virtual patient simulation systems
Simsek‐Cetinkaya (2023) [23]	AI‐assisted simulation practice	Traditional teaching model	20 min	②④	Virtual patient simulation systems
Fung et al. (2025) [24]	Interactive history‐taking between students and AI‐simulated patients	Immersive 360° VR video learning	120 min	②	Virtual patient simulation systems
Kestel et al. (2025) [25]	Traditional teaching + AI chatbot (Zeynep) for simulated history‐taking training	Traditional teaching model	2 weeks	②	Virtual patient simulation systems
Han et al. (2022) [26]	Interactive learning via video combined with AI chatbot	Video instruction	2 h	①③④	Conversational large language models
Liaw et al. (2024) [27]	Traditional simulation + AI virtual reality simulation	Traditional teaching model	3 h	①⑥	Virtual patient simulation systems
Başaran et al. (2024) [28]	Group 1: AI‐assisted instruction (ChatGPT) Group 2: Kahoot! interactive teaching platform	Traditional teaching model	2 weeks	①	Conversational large language models

*Note:* ① Knowledge level ② Practical competence ③ Critical thinking and clinical reasoning ability ④ Satisfaction ⑤ Attitude toward artificial intelligence ⑥ Communication skills ⑦ Humanistic care competence ⑧ Ethical awareness/decision‐making ⑨ Comprehensibility and operability ⑩ Digital literacy.

Abbreviations: QE, quasi‐experiment; RCT, randomized controlled trial.

## 3. Results

### 3.1. Literature Search Results

**FIGURE 1:**
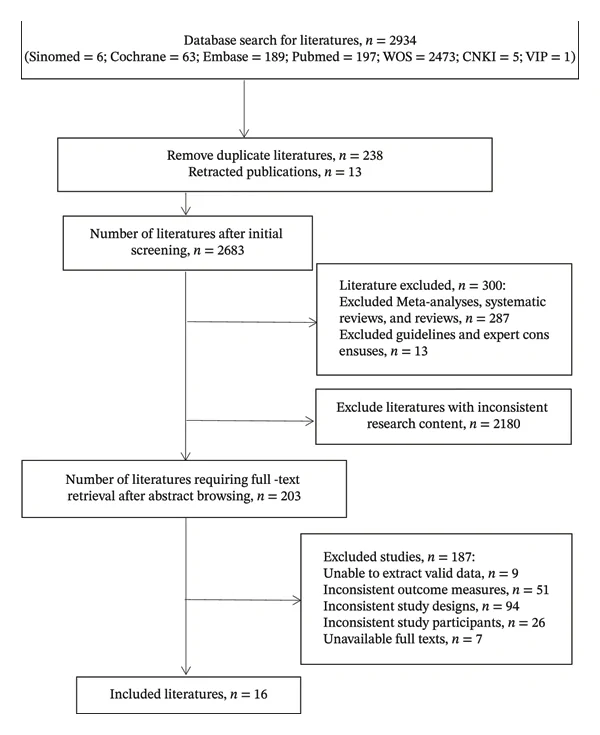
Study flowchart.

Through the literature search, 2934 articles in all—2922 in English and 12 in Chinese—were found. There were 2683 articles left after duplicates and retracted articles were eliminated. After examining the titles and abstracts of 2180 more studies, 300 more articles—including meta‐analyses, systematic reviews, and guidelines—were eliminated. After 203 articles′ full texts were acquired for additional screening, 16 articles were eventually included. The literature screening process and results are shown in Figure 1.

### 3.2. Basic Characteristics of Included Studies

This analysis comprised 16 publications published between 2023 and 2025, comprising 9 quasi‐experimental investigations and 7 RCTs. There were 1647 people in the entire sample. The basic characteristics of the included literature are presented in Table 2.

### 3.3. Methodological Quality Assessment Results of Included Studies

Seven RCTs were included in this study, all of which presented some concerns (see Table 3 for details). Among the 9 quasi‐experimental studies, 3 were Grade A and 6 were Grade B (see Table 4 for details).

**TABLE 3:** Methodological quality assessment of included RCTs (*n* = 7).

Included studies	Randomization process	Deviations from intervention	Missing outcome data	Outcome measurement	Selective reporting	Overall risk of bias
Guo et al. [7]	Low risk of bias	Some concerns	Low risk of bias	Low risk of bias	Low risk of bias	Some concerns
Saatçi et al. [9]	Low risk of bias	Some concerns	Low risk of bias	Some concerns	Low risk of bias	Some concerns
Arkan et al. [17]	Low risk of bias	Some concerns	Low risk of bias	Low risk of bias	Low risk of bias	Some concerns
Zhao and Zang [20]	Low risk of bias	Some concerns	Low risk of bias	Low risk of bias	Low risk of bias	Some concerns
Simsek‐Cetinkaya and Cakir [23]	Low risk of bias	Some concerns	Low risk of bias	Some concerns	Low risk of bias	Some concerns
Fung et al. [24]	Low risk of bias	Some concerns	Low risk of bias	Some concerns	Low risk of bias	Some concerns
Kestel et al. [25]	Low risk of bias	Some concerns	Low risk of bias	Low risk of bias	Low risk of bias	Some concerns

**TABLE 4:** Methodological quality assessment of included quasi‐experimental studies (*n* = 9).

Included studies	1	2	3	4	5	6	7	8	9	Quality level
Shin et al. [8]	Yes	Yes	Yes	Yes	No	No	Yes	Yes	Yes	B
Abuadas et al. [18]	Yes	Yes	Yes	Yes	Yes	No	Yes	Yes	Yes	B
Li et al. [19]	Yes	Yes	Yes	Yes	Yes	Yes	Yes	Yes	Yes	A
Molu [21]	Yes	Yes	Yes	Yes	Yes	Yes	Yes	Yes	Yes	A
Song et al. [10]	Yes	Yes	Yes	Yes	No	No	Yes	Yes	Yes	B
Park and Kim [22]	Yes	Yes	Yes	Yes	Yes	No	Yes	Yes	Yes	B
Han et al. [26]	Yes	Yes	Yes	Yes	Yes	No	Yes	Yes	Yes	B
Liaw et al. [27]	Yes	Yes	Yes	Yes	No	No	Yes	No	Yes	B
Başaran and Duru [28]	Yes	Yes	Yes	Yes	Yes	Yes	Yes	Yes	Yes	A

*Note:* (1) Whether the causal relationship is clearly stated; (2) Whether the baseline characteristics between groups are comparable; (3) Whether all other conditions/treatments are identical across groups except for the intervention; (4) Whether a control group is established; (5) Whether outcome measures are assessed using multiple indicators before and after the intervention; (6) Whether follow‐up is complete; (7) Whether outcome measures are assessed using the same methods across all groups; (8) Whether the measurement methods for outcome indicators are reliable; (9) Whether the data analysis methods are appropriate.

### 3.4. Meta‐Analysis Results

This study systematically evaluated the effects of applying AI in nursing practice courses through a meta‐analysis, and the main results are summarized in Table 5.

**TABLE 5:** Meta‐analysis results of the application effect of AI technology in nursing practice courses.

Outcome indicators	No. of included studies	Heterogeneity		Effect model	Meta‐analysis results			Grade evidence assessment
		*p*	*I* ^2^ (%)		SMD	95% CI	*p*	
Knowledge level	8	< 0.01	91	Random	0.72	0.20–1.24	< 0.01	Moderate
Practical competence	6	< 0.01	95	Random	1.25	0.50–2.00	< 0.01	Low
Critical thinking and clinical reasoning ability	4	0.41	0	Fixed	0.43	0.21–0.65	< 0.01	Very low
Satisfaction	3	0.26	26	Fixed	0.38	0.16–0.59	< 0.01	High
Ethical awareness/decision‐making	2	0.89	0	Fixed	0.50	0.29–0.71	< 0.01	Very low

#### 3.4.1. The Impact of AI Technology Application in Nursing Practice Courses on Students’ Knowledge Level

Eight studies [7, 19–22, 26–28] examined how students′ knowledge levels were affected by AI technology used in nursing practice courses. A random‐effects model was employed for the meta‐analysis since there was significant heterogeneity among the trials (*I* ^2^ = 91%, *p* < 0.001). According to the findings, students in the experimental group knew more than those in the control group, and this difference was statistically significant (SMD = 0.72, 95% CI [0.20–1.24], *p* = 0.007).

#### 3.4.2. Effect of AI Technology Application on Students’ Practical Ability in Nursing Practice Courses

Six studies [8, 17, 22–25] examined how the use of AI technology affected students′ practical ability in nursing practice courses. A random‐effects model was employed for the meta‐analysis since there was significant heterogeneity among the studies (*I* ^2^ = 95%, *p* < 0.001). The findings suggested that teaching nursing practice with AI assistance could improve students’ practical skills (SMD = 1.25, 95% CI [0.50–2.00], *p* = 0.001).

#### 3.4.3. Effects of AI Technology Application in Nursing Practice Courses on Students’ Critical Thinking and Clinical Reasoning Skills

Four studies [10, 19, 22, 26] examined how students′ critical thinking and clinical reasoning abilities were affected by the use of AI technology in nursing practice courses. Since there was no evidence of heterogeneity between the trials (*I* ^2^ = 0%, *p* = 0.41), the meta‐analysis employed a fixed‐effects model. The results showed that students in the experimental group demonstrated a slight advantage in critical thinking and clinical reasoning abilities compared with those in the control group, with a statistically significant difference (SMD = 0.43, 95% CI [0.21–0.65], *p* < 0.001).

#### 3.4.4. Effects of AI Technology Application in Nursing Practice Courses on Student Satisfaction and Ethical Awareness/Decision‐Making

Three studies [20, 23, 26] and two studies [10, 18] investigated the effects of AI technology application in nursing practice courses on student satisfaction and ethical awareness/decision‐making, respectively. No or mild heterogeneity was observed across studies (*I* ^2^ < 50%, *p* > 0.1); therefore, a fixed‐effects model was used for the meta‐analysis. The results showed that students in the experimental group showed a tendency toward higher satisfaction and ethical awareness/decision‐making ability compared with the control group, with statistically significant differences: (SMD = 0.38, 95% CI [0.16–0.59], *p* = 0.005), (SMD = 0.50, 95% CI [0.29–0.71], *p* < 0.001).

#### 3.4.5. Students’ Overall Attitudes Toward the Application of AI Technology in Nursing Practical Courses

Only two studies [17, 18] explored nursing students’ positive and negative attitudes toward AI implementation in practical nursing courses. Given the insufficient sample size and high heterogeneity for meta‐analysis, findings were summarized narratively. For positive attitudes, both trials reported superior outcomes in the intervention versus control groups: Arkan et al. reported an SMD of 0.69 (95% CI [0.27–1.10]), and Abuadas et al. yielded an SMD of 1.39 (95% CI [0.98–1.80]). Regarding negative attitudes, Arkan et al. found significantly lower negative sentiment among intervention‐group students (SMD = 0.56, 95% CI [0.15–0.97]), whereas Abuadas et al. observed a smaller, nonsignificant effect size (SMD = 0.32; 95% CI [−0.05–0.69]). Since this synthesis draws exclusively from two original studies, the available evidence remains limited, and further high‐quality investigations are required to corroborate these results.

#### 3.4.6. Results of Subgroup Analyses for Knowledge Level and Practical Ability

Subgroup analyses stratified by research design, intervention duration, and kind of AI tool were carried out in this meta‐analysis to further investigate potential sources of significant variability in knowledge level and practical ability outcomes (Tables 6 and 7). For knowledge level, long‐duration interventions (more than 1 week) yielded moderate improvement effects (SMD = 0.95, 95% CI [0.20–1.70]), while no statistically meaningful differences were detected in short‐duration intervention subgroups. Stratification by study design revealed that AI interventions appeared beneficial within the RCT subgroup (SMD = 1.08, 95% CI [0.13–2.04]). In contrast, quasi‐experimental studies failed to demonstrate a clear statistically significant effect (SMD = 0.60, 95% CI [−0.07–1.27]). Substantial heterogeneity persisted across both subgroups, indicating that study design may not serve as a primary contributor to overall heterogeneity. Regarding practical ability outcomes, long‐duration interventions tended to produce stronger effects relative to short‐duration interventions (SMD = 1.72 versus 0.30). Conversational large language models also exhibited a trend toward superior efficacy compared to virtual patient simulation systems (SMD = 1.67 versus −0.26). Stratified analyses by study design showed positive potential effects of AI interventions on practical ability in both RCTs (SMD = 1.15, 95% CI [0.20–2.10]) and quasi‐experimental studies (SMD = 1.57, 95% CI [1.22–1.91]). However, the quasi‐experimental subgroup only included two original studies, which warrants considerable caution when interpreting these findings.

**TABLE 6:** Subgroup analysis for knowledge level.

Subgroup	No. of included studies	Heterogeneity		Effect model	Effect size	95% CI
		*I* ^2^ (%)	*p*			
*Study design*						
RCT	2	92	< 0.01	Random	1.08	0.13–2.04
Quasi‐experimental study	6	92	< 0.01	Random	0.60	−0.07–1.27

*Intervention duration*						
Short‐term (< 1 week)	4	93	< 0.01	Random	0.50	−0.33–1.33
Long‐term (> 1 week)	4	91	< 0.01	Random	0.95	0.20–1.70

**TABLE 7:** Subgroup analysis of practical ability.

Subgroup	No. of included studies	Heterogeneity		Effect model	Effect size	95% CI
		*I* ^2^ (%)	*p*			
*Study design*						
RCT	4	96	< 0.01	Random	1.15	0.20–2.10
Quasi‐experimental study	2	44	0.18	Fixed	1.57	1.22–1.91

*Intervention duration*						
Short‐term (< 1 week)	3	97	< 0.01	Random	0.30	−1.23–1.82
Long‐term (> 1 week)	3	89	< 0.01	Random	1.72	1.10–2.34

*AI tools*						
Conversational large language models	4	86	< 0.01	Random	1.67	1.15–2.18
Virtual patient systems	2	94	< 0.01	Random	−0.26	−1.75–1.23

### 3.5. Publication Bias

When 10 or more qualifying studies are included, a publication bias evaluation is necessary. A funnel plot could not be created to assess publication bias due to the small number of trials combined for each outcome measure (all fewer than 10). The Discussion section goes into great length on this limitation as a major study constraint.

## 4. Discussion

The PRISMA 2020 standards were followed in the conduct of this systematic review and meta‐analysis. The efficiency of AI‐assisted training in practical coursework was assessed by combining data from 16 studies (7 RCTs and 9 quasi‐experimental studies) including 1647 nursing students. Pooled analyses showed positive results for several endpoints; nevertheless, significant heterogeneity and moderate‐to‐low certainty according to GRADE criteria call for careful interpretation of these findings. Methodological quality, key findings, and study limitations are covered in the sections that follow.

### 4.1. The Methodological Quality of the Included Studies Was Generally Above Average

The trustworthiness of the meta‐analysis results was established by the moderate to high overall methodological quality of the 16 publications (7 RCTS and 9 quasi‐experimental studies) included in this study. Of the nine quasi‐experimental investigations, three totally satisfied the JBI critical assessment criteria and were rated as Grade A, while the remaining six were rated as Grade B. All seven of them raised some issues. Some studies found a number of problems, such as a lack of varied assessments for outcome markers and insufficient follow‐up. All research assessed teaching effectiveness using standardized outcome measures, despite the fact that the AI tools (e.g., ChatGPT, DeepSeek, Danbee.ai) and teaching durations varied across studies, potentially introducing some clinical heterogeneity. Additionally, two researchers independently screened the literature and evaluated its quality, which lessened subjective bias. To lower heterogeneity and increase the trustworthiness of outcomes, future research should improve follow‐up procedures, tighten blinding designs, and standardize fundamental outcome measurements.

### 4.2. Application of AI Technology in Nursing Practical Teaching to Improve Students’ Knowledge Level and Practical Ability

Results of the present meta‐analysis indicated that integrating AI into nursing practical curricula yields positive improvements in nursing students’ theoretical knowledge (SMD = 0.72) and clinical operational skills (SMD = 1.25). Solid theoretical knowledge serves as the foundation for ensuring clinical safety and cultivating professional competency. Insufficient theoretical proficiency tends to trigger adverse incidents including medication administration errors, biased condition assessments, and delayed clinical decision‐making. This meta‐analysis confirms that students can build systematic knowledge architectures with the aid of AI‐enabled knowledge graphs, intelligent Q&A systems, and targeted knowledge interpretation. It also addresses a major drawback of traditional pedagogy, which is the absence of real‐time guidance when students face challenging learning problems. AI‐assisted simulation training, on the other hand, relieves the burden on limited clinical internship resources by enabling students to hone their practical ability in the face of limited clinical placement possibilities. Academic success in nursing coursework and clinical practice outcomes are eventually optimized by the concurrent development of students’ theoretical foundation and practical ability.

Subgroup analyses focused on knowledge level indicate that intervention duration may serve as a critical moderator of intervention effects. Longer interventions (more than 1 week) yielded a larger pooled effect size (SMD = 0.95, 95% CI [0.20–1.70]) compared with short‐term interventions (less than 1 week) (SMD = 0.50, 95% CI (−0.33–1.33]). Mechanistically, AI overcomes the “one‐size‐fits‐all” and “no‐opportunity‐to‐ask‐questions” limitations of traditional classrooms through intelligent content generation and personalized learning support, and facilitates knowledge internalization via real‐time Q&A and knowledge graphs. Relevant studies [20, 26] also indicate that AI‐assisted practical instruction can promptly correct cognitive biases and enhance learning outcomes. At the practical ability, subgroup analyses also suggest that intervention duration and the type of AI tools serve as key moderators influencing intervention efficacy. Long‐term interventions (more than 1 week) yielded a more pronounced positive trend relative to short‐term interventions (less than 1 week) (SMD = 1.72 vs. 0.30). This finding implies that the acquisition and consolidation of practical ability require prolonged repeated practice and timely feedback. In terms of tool categories, pooled effect sizes were larger for conversational large language models (SMD = 1.67) compared with virtual patient systems (SMD = −0.26). The ability of conversational large language models to imitate dynamic, open‐ended clinical dialogs and provide learners with real‐time feedback may be the cause of this. The drawbacks of traditional education, such as the lack of standardized patients, the scarcity of clinical practice chances, and the delayed feedback, can be made up for by AI‐assisted simulation instruction. It gives students safe, consistent training settings to enhance their communication, history‐taking, and nursing operations abilities [24, 29]. This teaching methodology transforms passive knowledge receipt into inquiry‐based active learning, fulfills personalized learning demands, and eases the load on clinical teaching resources. It enables students establish sufficient skill reserves before to real‐world clinical internships, breaks the restrictions of traditional education from the dimensions of cognitive building and skill acquisition, and achieves simultaneous improvement in knowledge and practical competence [30].

Although the aforementioned findings are positive, substantial statistical heterogeneity was identified across both outcome measures. Subgroup analysis identified intervention length and the type of AI technology as possible factors. Nevertheless, unexplained residual heterogeneity shows that additional confounding factors—such as changes in instructional environments and baseline characteristics of learners—also contribute to variances in results among particular research. Potential contributory factors are summarized as follows. First, different studies have given AI different pedagogical roles. In some, AI is used as an autonomous tutoring agent; in others, it is incorporated into official curriculum instruction; in still others, it is only used as a pre‐class preparation tool. Heterogeneous learning results are unavoidably the result of these different instructional roles, but the original literature lacks specific information to enable focused quantitative study. Second, there are significant differences in the tools used to measure outcomes. While skill evaluations use faculty rating checklists, custom‐built scales, and Objective Structured Clinical Examinations (OSCE), knowledge assessments use self‐designed test papers, standardized exams, and clinical scenario‐based assessments. There are significant dangers of measurement bias because the majority of these evaluation instruments do not record reliability and validity data. Third, subgroup analyses stratified by these baseline indicators are rarely carried out in published studies, despite the fact that student baseline characteristics—such as prior academic knowledge reserves, AI literacy, and practical clinical experience—are diverse between participant cohorts. Last but not least, there are significant differences between institutions and countries in contextual factors including class size, practical training hours, and faculty training programs for AI literacy, which may further modify the extent of intervention benefits. These factors together make up the main causes of study heterogeneity. However, comprehensive, objective dissection and analysis of such variability is impossible due to varied reporting standards across various studies. As a result, care should be taken when interpreting these pooled effect estimates. Standardized intervention protocols and basic outcome markers should be used in future research to increase cross‐study comparability and decrease between‐study variability.

### 4.3. Application of AI Technology in Nursing Practice Education to Improve Students’ Critical Thinking and Clinical Reasoning Skills

The findings of this meta‐analysis showed that students′ critical thinking and clinical reasoning skills can be greatly enhanced by incorporating AI into nursing practice education. Additional examination of its mechanism shows that the structured and directed application of AI is the key to its beneficial effects: AI first relieves pupils of the effort of retrieving knowledge, freeing up more cognitive resources for higher‐order thinking tasks. Second, AI promotes ongoing reflection through multiround engagement, which enhances students’ comprehension of clinical circumstances. Lastly, the multifaceted reference data that AI offers enable pupils to pinpoint their blind areas and improve their thought processes. Although the development in critical thinking skills (SMD = 0.43) only produces a moderate effect size from a clinical safety standpoint, its therapeutic importance cannot be disregarded. Modest improvements in clinical reasoning help reduce therapeutic and diagnostic errors in high‐risk clinical settings. These results imply that AI‐augmented instruction can promote active reflection, standardize clinical decision‐making workflows, and aid in the development of safer frameworks for clinical practice when trainees receive focused training to critically assess outputs produced by AI rather than accepting such content indiscriminately.

Unsupervised or over‐reliance on AI, however, may have unfavorable consequences. First, there is a greater chance of automated bias. According to research by Lazarus et al., students frequently follow AI‐generated clinical recommendations without question, which could cause doctors to overlook clinical indicators and implement incorrect treatment plans in clinical decision‐making systems [31]. Second, due to faulty data sources and delayed knowledge upgrades, AI frequently generates unclear explanations and out‐of‐date nursing guidelines. Nursing students may form improper clinical judgments based on erroneous results if they lack proficient critical assessment skills. Finally, skill atrophy is a result of long‐term reliance on AI. Participating nursing students noted that fast access to answers through AI diminishes autonomous inquiry and in‐depth reasoning; excessive use further hinders their ability to communicate verbally and in writing [32]. Students’ capacity for autonomous reasoning will be limited, and their ability to make independent decisions in difficult situations will progressively decline, if they see AI as a quick fix for thinking and do not engage in critical analysis [33]. A collaborative model combining AI and human instruction should be investigated in nursing education in the future. It should foster students’ critical thinking and autonomous clinical decision‐making abilities while utilizing AI’s benefits.

### 4.4. The Application of AI Technology in Nursing Practice Teaching has Positive Effects on Student Satisfaction and Ethical Awareness/Decision‐Making

The use of AI in nursing practice courses enhances students’ learning satisfaction, ethical awareness, and decision‐making skills, according to this meta‐analysis. In terms of improving learning satisfaction, AI delivers a better learning experience than traditional teaching by enhancing learner autonomy and engagement, while offsetting insufficient practical opportunities and delayed feedback [34]. AI can provide role‐playing and real‐time decision‐making feedback while simulating real‐world ethical issues to foster ethical awareness and decision‐making ability. This effectively overcomes the “paper‐only” constraints of traditional training and advances students’ ethical awareness by enabling them to exercise frequently in a secure setting [35].

In the GRADE certainty ratings, the evidence for satisfaction was graded as high certainty. The three pooled studies exhibited low statistical heterogeneity (*I* ^2^ = 26%), consistent direction of effect estimates, and relatively low overall risk of bias, with limited study volume being the sole limitation. The evidence for knowledge level and practical ability, on the other hand, was rated as having moderate‐to‐low certainty. This was primarily due to significant statistical heterogeneity (*I* ^2^ > 90%) and methodological issues among the included studies, such as incomplete follow‐up data and the lack of blinding in quasi‐experimental designs. Due to the reliance on only two to four small‐sample studies, the evidence for ethical awareness and critical thinking was graded as having very low certainty; also, formal evaluation of publication bias was not possible due to the short number of included studies (*n* < 10). The preliminary nature of the current data is highlighted by these assessments, which also emphasize the urgent need for carefully planned primary investigations to increase the reliability of pooled impact estimates.

However, incorporating AI into practical nursing education also brings up a number of ethical and practical issues that demand careful consideration. First, there are significant dangers related to data security and privacy: most commercial platforms lack transparent data governance, which leaves holes in the control of data storage and utilization, and students may divulge private information when using AI [36]. Second, irresponsible over‐reliance by learners might result in the retention of faulty knowledge and ultimately jeopardize patient safety because AI‐generated content is prone to errors or incorrect outputs [37]. Third, there are still significant digital gaps in access to AI resources since free or entry‐level software has practical restrictions and could exacerbate educational inequality [38]. Educators should create uniform ethical standards, bolster data security procedures, raise staff and student AI knowledge, and encourage fair access to these tools.

### 4.5. Students’ Attitudes Toward the Application of AI in Nursing Practice Teaching Require Further Research and Validation

The results of this study showed that nursing students typically have favorable opinions about the use of AI in nursing practice courses, indicating that nursing students have acknowledged and embraced AI‐assisted practice education. This optimistic outlook might result from the enhanced educational experience that AI offers. However, there was significant heterogeneity and only two studies were considered for this outcome measure. This could be explained by variations in the kinds of AI tools and course material used in different research. In order to give a reference for the promotion and use of AI in nursing education, more excellent study should be carried out in the future to better investigate the major aspects impacting students’ perspectives.

### 4.6. Limitations of the Study

In order to examine the efficacy of AI in nursing practice courses, this study meticulously adhered to systematic review methodologies to analyze the included literature and quantitatively synthesize outcome metrics. There are still a number of restrictions, despite the fact that it somewhat expanded the sample size and enhanced statistical power: ① Every 1 of the 16 RCTs that were included of this review had some quality issues. Because of the intrinsic nature of the therapies, the primary restriction was the inability to blind participants and intervention providers. The strength of the evidence may have been impacted by problems with several quasi‐experimental studies, such as nondiverse outcome measuring and inadequate follow‐up. ② A number of outcome markers showed moderate‐to‐high heterogeneity due to significant differences in intervention length, course content, and outcome assessment instruments between trials. The stability of pooled data may still be jeopardized even after modification using the random‐effects model. This study’s categorization of AI tools and data pooling techniques is another drawback. This study combined and examined conversational large language models and virtual‐patient simulation systems, which have quite different operational mechanisms. While the latter develops students′ reasoning abilities through interactive discussion and feedback, the former concentrates on practical decision‐making training inside simulated clinical scenarios. Table 7’s subgroup analyses show that combining these two kinds of tools could increase overall heterogeneity and obscure the actual impact of particular interventions. ③ This study was unable to do a thorough assessment of publication bias since less than 10 studies were considered for each particular outcome indicator. When assessing pooled impact sizes, care should be taken because this could exaggerate the effects of AI‐based therapies and over‐represent positive outcomes. Nine quasi‐experimental papers were also included in this meta‐analysis. Numerous biases, such as baseline imbalance and participants’ spontaneous physiological or behavioral changes, can affect such nonrandomized investigations. Due to insufficient follow‐up and few outcome measures, six of these nine quasi‐experimental studies received a level B evidence rating. These methodological flaws run the danger of overestimating the effects of interventions and lowering the dependability of pooled data. Robust and significant comparison analyses were not possible due to the small number of qualifying RCTs and the high risk of bias associated with existing RCTs, even though subgroup analyses were carried out to investigate effect differences across research designs. ④ Language bias may have been generated because only Chinese and English literature was included due to language restrictions. These results are still preliminary and need to be confirmed by more high‐caliber research because of restrictions on the quantity and methodological quality of included studies as well as the significant heterogeneity seen in a number of outcome indicators. Policymakers and nursing educators should objectively acknowledge the current state of the research base and interpret these pooled effect estimates with the necessary caution. The evidence of moderate to low certainty highlights the need for thorough additional research before implementing AI‐assisted teaching methods on a large scale.

## 5. Summary

In order to methodically assess the efficacy of AI in nursing practice courses, this study performed a systematic review and meta‐analysis of 16 studies. According to preliminary data, nursing students’ knowledge level, practical ability, critical thinking, clinical reasoning, learning satisfaction, ethical awareness, and decision‐making ability are all positively impacted by AI‐assisted practical instruction. Additionally, students’ attitudes regarding AI‐assisted instruction were usually good. The overall methodological quality of the included studies was moderate to high, but certain limitations remained. For instance, some studies failed to implement blinding, had incomplete follow‐up, or used inconsistent tools for outcome measurement, leading to high heterogeneity in some outcome indicators. Overall certainty of evidence ranged from moderate to low, and substantial heterogeneity observed for knowledge and practical outcomes limits interpretability of pooled estimates. Findings should therefore be interpreted cautiously. In the future, more rigorously designed, multicenter, and large‐sample studies should be conducted to unify core outcome measures, explore optimal pathways for the deep integration of AI into nursing education, and provide high‐quality evidence for the intelligent transformation of nursing education.

Implications for nursing management: Nursing education management can gain multifaceted practical insights from the use of AI in practical training courses: (1) Institutions shall actively adopt mechanisms for the implementation, evaluation, and continuous improvement of AI‐assisted teaching; allocate resources in a coordinated manner; and advance the intelligent upgrading of practical courses to ease the shortage of clinical practice resources. (2) To improve nursing educators’ competence in AI instructional design and ethical standards, a tiered and categorized training system will be implemented. To promote educational innovation, corresponding reward and assessment schemes should be developed. (3) Teaching management must incorporate a multifaceted evaluation system for AI teaching outcomes. Formative assessment using platform data can be used to continuously monitor and enhance the quality of instruction. (4) The goals of nursing talent training should include AI digital literacy. To avoid an over‐reliance on technology, instructors must cultivate students’ critical thinking and autonomous judgment while enhancing their practical skills. (5) To standardize educational data management, a strong ethical and safety framework for AI application must be refined. This ensures a uniform and long‐lasting intelligent change of nursing education by striking a balance between humanistic care and technology empowerment.

## Funding

This work was supported by the Special Project for Selecting High‐Level Scientific and Technological Talents and Innovation Team of Yunnan Province‐Technical Innovation Talent Training Target Project under Grant Number: 202305AD160007, Yunnan Health Training Project of High Level Talents (Number: D‐2024022), Applied Basic Research Joint Special Fund Project of Science and Technology Department of Yunnan Province, and Kunming Medical University (Number: 202201AY070001‐080).

## Conflicts of Interest

The authors declare no conflicts of interest.

## Supporting Information

Additional supporting information can be found online in the Supporting Information section.

## Supporting information


**Supporting Information** Figure S1: (Forest plot, file named Forest plot.docx). Table S1: (GRADE evidence summary, file named GRADE.docx). PRISMA 2020 checklist (file named PRISMA 2020 checklist.docx). These materials provide detailed analytical results and methodological quality assessment support for this meta‐analysis.

## Data Availability

The datasets analyzed in this study were obtained from published literature, with specific sources listed in the references. The extracted data used in this study are available in the tables within the main text.

## References

[1] Cucci F. , Marasciulo D. , Romani M. et al., The Contribution of Artificial Intelligence in Nursing Education: A Scoping Review of the Literature, Nursing Reports. (2025) 15, no. 8, 10.3390/nursrep15080283. PMC12389495 40863670

[2] Tischendorf T. , Hasseler M. , Schaal T. et al., Developing Digital Competencies of Nursing Professionals in Continuing Education and Training: A Scoping Review, Frontiers of Medicine. (2024) 11, 10.3389/fmed.2024.1358398. PMC11212473 38947234

[3] Ye Z. W. , Li X. W. , and Huang L. L. , Practice and Effect Evaluation of Anchored Instruction in Health Education Course for Undergraduate Nursing Students from the Perspective of Healthy China 2030, Frontiers in Medicine. (2025) 15, no. 33, 103–107, 10.20235/j.issn.2095-1752.2025.33.023.

[4] Gürdil Yilmaz S. , Yıldız Karadeniz E. , and dem Lafçi D. , Clinical‐Practice Stress Levels and Factors Affecting These on First‐Year Nursing Students, Perspectives in Psychiatric Care. (2022) 58, no. 4, 3009–3015, 10.1111/ppc.13070. 35318676

[5] De Gagne J. C. , The State of Artificial Intelligence in Nursing Education: Past, Present and Future Directions, International Journal of Environmental Research and Public Health. (2023) 20, no. 14, 10.3390/ijerph20064884. PMC10049425 36981790

[6] Sallam M. , ChatGPT Utility in Healthcare Education, Research, and Practice: Systematic Review on the Promising Perspectives and Valid Concerns, Healthcare. (2023) 11, no. 6, 10.3390/healthcare11060887. PMC10048148 36981544

[7] Guo Z. S. , Liu Y. L. , Xu Y. , and Chen X. , Application of a GAI-Enabled Interdisciplinary Collaborative Learning Model in Practical Teaching of Nursing Psychology Among Undergraduate Nursing Students, Zhonghua Xian Dai Hu Li Za Zhi. (2025) 31, no. 28, 3889–3893, 10.3760/cma.j.cn115682-20250602-02883.

[8] Shin H. , De Gagne J. , Kim S. , and Hong M. , The Impact of Artificial intelligence-assisted Learning on Nursing Students’ Ethical Decision-Making and Clinical Reasoning in Pediatric Care: A Quasi-Experimental Study, Computers Informatics Nursing. (2024) 42, no. 10, 704–711, 10.1097/CIN.0000000000001177. PMC11458082 39152099

[9] Saatçi G. , Korkut S. , and Ünsal A. , The Effect of the Use of Artificial Intelligence in the Preparation of Patient Education Materials by Nursing Students on the Understandability, Actionability and Quality of the Material: A Randomized Controlled Trial, Nurse Education in Practice. (2024) 81, 10.1016/j.nepr.2024.104186. 39520840

[10] Song D. , Zhang P. , Zhu Y. et al., Effects of Generative Artificial Intelligence on Higher-Order Thinking Skills and Artificial Intelligence Literacy in Nursing Undergraduates: A Quasi-Experimental Study, Nurse Education in Practice. (2025) 88, 10.1016/j.nepr.2025.104549. 40915080

[11] Ni C. P. , Li L. , and Liu X. W. , Application and Experience of Constructivist Learning Theory in Continuing Nursing Education, Journal of Nursing Administration. (2012) 12, no. 4, 288–289.

[12] McCoy L. G. , Ci Ng F. Y. , Sauer C. M. et al., Understanding and Training for the Impact of Large Language Models and Artificial Intelligence in Healthcare Practice: A Narrative Review, BMC Medical Education. (2024) 24, no. 1, 10.1186/s12909-024-06048-z. PMC11459854 39375721

[13] El Arab R. A. , Al Moosa O. A. , Abudass F. H. , and Somerville J. , The Role of AI in Nursing Education and Practice: Umbrella Review, Journal of Medical Internet Research. (2025) 27, 10.2196/69881. PMC12008698 40072926

[14] Buchanan C. , Howitt M. L. , Wilson R. , Booth R. G. , Risling T. , and Bamford M. , Predicted Influences of Artificial Intelligence on the Domains of Nursing: Scoping Review, JMIR Nurs. (2020) 3, no. 1, 10.2196/23939. PMC8373374 34406963

[15] Sterne J. A. C. , Savović J. , Page M. J. et al., RoB 2: A Revised Tool for Assessing Risk of Bias in Randomised Trials, BMJ. (2019) 28, 10.1136/bmj.l4898. 31462531

[16] Munn Z. , Barker T. H. , Moola S. et al., Methodological Quality of Case Series Studies: An Introduction to the JBI Critical Appraisal Tool, JBI Database of Systematic Reviews and Implementation Reports. (2020) 18, no. 10, 2127–2133, 10.11124/JBISRIR-D-19-00099. 33038125

[17] Arkan B. , Dallı Ö. E. , and Varol B. , The Impact of ChatGPT Training in the Nursing Process on Nursing Students’ Problem-Solving Skills, Attitudes Towards Artificial Intelligence, Competency, and Satisfaction Levels: Single-Blind Randomized Controlled Study, Nurse Education Today. (2025) 152, 10.1016/j.nedt.2025.106765. 40334550

[18] Abuadas M. , Albikawi Z. , and Rayani A. , The Impact of an AI-Focused Ethics Education Program on Nursing Students’ Ethical Awareness, Moral Sensitivity, Attitudes, and Generative AI Adoption Intention: A Quasi-Experimental Study, BMC Nursing. (2025) 24, no. 1, 10.1186/s12912-025-03458-2. PMC12211453 40597065

[19] Li C. , Hwang G. , Chang C. , and Su H. , Generative AI-Supported Progressive Prompting for Professional Training: Effects on Learning Achievement, Critical Thinking, and Cognitive Load, British Journal of Educational Technology. (2025) 56, no. 6, 2550–2572, 10.1111/bjet.13594.

[20] Zhao J. and Zang M. , Exploring the Integration of AI and National Quality Courses in China: A Study on Teaching Practices in Nursing Smart Education, BMC Medical Education. (2025) 25, no. 1, 10.1186/s12909-025-08289-y. PMC12751411 41466245

[21] Molu B. , Improving Nursing Students’ Learning Outcomes in Neonatal Resuscitation: A Quasi-Experimental Study Comparing AI-Assisted Care Plan Learning With Traditional Instruction, Journal of Evaluation in Clinical Practice. (2025) 31, no. 1, 10.1111/jep.14286. 39733257

[22] Park S. A. and Kim H. Y. , Development and Effects of a Scenario-Based Labor Nursing Simulation Education Program Using an Artificial Intelligence Tutor: A Quasi-Experimental Study, Women′s Health Nursing. (2025) 31, no. 2, 143–154, 10.4069/whn.2025.06.18. PMC12245539 40639863

[23] Simsek-Cetinkaya S. and Cakir S. , Evaluation of the Effectiveness of Artificial Intelligence Assisted Interactive Screen-Based Simulation in Breast Self-Examination: An Innovative Approach in Nursing Students, Nurse Education Today. (2023) 127, 10.1016/j.nedt.2023.105857. 37253303

[24] Fung T. , Chan S. , Lam C. et al., Effects of Generative Artificial Intelligence (Genai) Patient Simulation on Perceived Clinical Competency Among Global Nursing Undergraduates: A Cross-Over Randomised Controlled Trial, BMC Nursing. (2025) 24, no. 1, 10.1186/s12912-025-03492-0. PMC12272990 40676632

[25] Kestel S. , Çalık A. , and Kuş M. , The Effect of Chatbot-Supported Instruction on Nursing Students’ History-Taking Questioning Skills and Stress Level: A Randomized Controlled Study, Journal of Professional Nursing. (2025) 60, 93–100, 10.1016/j.profnurs.2025.07.004. 40915772

[26] Han J. W. , Park J. , and Lee H. , Analysis of the Effect of an Artificial Intelligence Chatbot Educational Program on Non-Face-to-Face Classes: A Quasi-Experimental Study, BMC Medical Education. (2022) 22, no. 1, 10.1186/s12909-022-03898-3. PMC9713176 36457086

[27] Liaw S. Y. , Rusli K. D. B. , Tan J. Z. , Wee Y. H. C. , Neo N. W. S. , and Chua W. L. , Artificial Intelligence-Enabled Virtual Reality Simulation for Clinical Deterioration Training: An Effectiveness-Implementation Hybrid Study, Nurse Education in Practice. (2025) 87, 10.1016/j.nepr.2025.104462. 40627957

[28] Basaran F. and Duru P. , The Impact of Kahoot and ChatGPT Educational Technologies on Nursing Students’ Sexual Health Knowledge and Attitudes: A Quasi-Experimental Study, Sexuality and Disability. (2024) 42, no. 4, 801–815, 10.1007/s11195-024-09873-8.

[29] Jembu J. P. and Lee S. , The Effectiveness of Artificial Intelligence (AI) in Learning Outcomes of Nursing Students: A Meta-Analysis of Randomized Controlled Trials (RCTs), STEM Education. (2025) 5, no. 4, 546–563, 10.3934/steme.2025026.

[30] Yin Y. and Liu S. Y. , Innovation of Nursing Talent Training Model in Universities Empowered by Artificial Intelligence, East China University of Science and Technology. (2025) no. 09, 138–140.

[31] Lazarus M. D. , Truong M. , Douglas P. , and Selwyn N. , Artificial Intelligence and Clinical Anatomical Education: Promises and Perils, Anatomical Sciences Education. (2024) 17, no. 2, 249–262, 10.1002/ase.2221. 36030525

[32] Hu R. J. , Mu Y. Y. , and Leng L. L. , A Qualitative Study on Generative Artificial Intelligence Literacy Among Nursing Undergraduates Based on the NURSES Competency Framework, Frontiers in Medicine. (2025) 15, no. 34, 11–15, 10.20235/j.issn.2095-1752.2025.34.003.

[33] Din A. L. , How AI Tools (Like ChatGPT/Gemini) Affect Critical Thinking in Nursing Students: A Systematic Review, Journal of Health Science and Medical Research. (2026) 3, no. 1, 10.65035/650ytk03.

[34] Peng J. , Zhang H. , Tu X. et al., Effectiveness of AI-Assisted Medical Education for Chinese Undergraduate Medical Students: A Meta-Analysis, BMC Medical Education. (2025) 25, no. 1, 10.1186/s12909-025-07770-y. PMC12382127 40866973

[35] Reed A. , Andrews A. , and Frost E. , Undergraduate Nursing Students’ Experiences With Artificial Intelligence in a Research and Evidence-Based Practice Course, CIN: Computers, Informatics, Nursing. (2025) 10, no. 5, 10.1097/CIN.0000000000001419. 41432084

[36] Bozkurt S. A. , Aydoğan S. , Dursun Ergezen F. , and Türkoğlu A. , A Systematic Review and Sequential Explanatory Synthesis: Artificial Intelligence in Healthcare Education: A Case of Nursing, International Nursing Review. (2025) 72, no. 2, 10.1111/inr.70018. PMC12005066 40243390

[37] Liu J. , Liu F. , Fang J. , and Liu S. , The Application of Chat Generative Pre-Trained Transformer in Nursing Education, Nursing Outlook. (2023) 71, no. 6, 10.1016/j.outlook.2023.102064. 37879261

[38] Yasin Y. M. , Al‐Hamad A. , Metersky K. , and Kehyayan V. , Incorporation of Artificial Intelligence Into Nursing Research: A Scoping Review, International Nursing Review. (2025) 72, no. 1, 10.1111/inr.13013. PMC11741909 38967044

