# Supplementary material for: Artificial Intelligence in Nursing Practice Education: A Systematic Review and Meta‐Analysis
Source: J Nurs Manag. 2026 Sep 10;2026:6626865. doi: 10.1155/jonm/6626865 (PMC13562914; doi:10.1155/jonm/6626865)
Supplement: Supplementary file 1 [file JONM-2026-6626865-s001.zip › Forest plot.docx]

Knowledge Level：

Practical Ability：

Critical Thinking and Clinical Reasoning Skills：

Satisfaction：

Ethical Awareness/Decision-Making：

**Subgroup Analysis by Level of Knowledge:**

1.Study Design (RCT vs. Quasi-Experiment)

RCT

Quasi-Experiment

2.Duration of Intervention (Short-Term vs. Long-Term)

Short-Term（＜1 week）

Long-Term（＞1 week）

**Subgroup Analysis of Practical Competence:**

1.Study Design (RCT vs. Quasi-Experiment)

RCT

Quasi-Experiment

2.Duration of Intervention (Short-Term vs. Long-Term)

Short-Term（＜1 week）

Long-Term（＞1 week）

3.AI Tools（Conversational Large Language Models vs. Virtual Patient Systems）

Conversational Large Language Models

Virtual Patient Systems
