# Supplementary material for: Artificial Intelligence in Nursing Practice Education: A Systematic Review and Meta‐Analysis
Source: J Nurs Manag. 2026 Sep 10;2026:6626865. doi: 10.1155/jonm/6626865 (PMC13562914; doi:10.1155/jonm/6626865)
Supplement: Supplementary file 1 [file JONM-2026-6626865-s001.zip › GRADE.docx]

**Author(s):**

**Question:** [
] c ompared to [
] for [

] **Setting:**

**Bibliography:**

| **Certainty assessment** | | | | | | | **№ of patients** | | **Effect** | | **Certainty** | **Importance** |
| --- | --- | --- | --- | --- | --- | --- | --- | --- | --- | --- | --- | --- |
| **№ of**  **studies** | **Study design** | **Risk of bias** | **Inconsistency** | **Indirectness** | **Imprecision** | **Other considerations** | **[****]** | **[****]** | **Relative**  **(95% CI)** | **Absolute**  **(95% CI)** |  |  |

| 8 | randomised  trials | seriousa | not serious | not serious | not serious | none | 377 | 391 | - | S MD **0.72**  **higher**  (0 .2 higher to  1 .24 higher) | ⨁⨁⨁◯  Moderatea |  |
| --- | --- | --- | --- | --- | --- | --- | --- | --- | --- | --- | --- | --- |

| 6 | randomised  trials | seriousa | seriousb | not serious | not serious | none | 374 | 373 | - | S MD **1.25**  **higher**  (0 .5 higher to  2 higher) | ⨁⨁◯◯  Lowa,b |  |
| --- | --- | --- | --- | --- | --- | --- | --- | --- | --- | --- | --- | --- |

| 4 | non-  randomised  studies | seriousa | seriousc | not serious | not serious | none | 166 | 161 | - | S MD **0.43**  **higher**  (0 .21 higher  to 0 .65  higher) | ⨁◯◯◯  Very lowa,c |  |
| --- | --- | --- | --- | --- | --- | --- | --- | --- | --- | --- | --- | --- |

| 3 | randomised  trials | not serious | not serious | not serious | not serious | none | 178 | 171 | - | S MD **0.38**  **higher**  (0 .16 higher  to 0 .59  higher) | ⨁⨁⨁⨁  High |  |
| --- | --- | --- | --- | --- | --- | --- | --- | --- | --- | --- | --- | --- |

**/**

| 2 | non-  randomised  studies | seriousa | not serious | not serious | not serious | none | 180 | 182 | - | S MD **0.5**  **higher**  (0 .29 higher  to 0 .71  higher) | ⨁◯◯◯  Very lowa |  |
| --- | --- | --- | --- | --- | --- | --- | --- | --- | --- | --- | --- | --- |

**CI:** confidence interval; **SMD:** standardised mean difference **Explanations**

a .

b .

AI

 c .
